# A novel and effective surgical procedure for posterior nasal neurectomy in patients with allergic rhinitis

**DOI:** 10.1016/j.bjorl.2025.101653

**Published:** 2025-05-27

**Authors:** Weini Hu, Qiang Zuo, Dawei Wu, Yu Song

**Affiliations:** a Peking University Third Hospital, Department of Otorhinolaryngology Head and Neck Surgery, Beijing, China; b Yan’an Traditionnal Chinese Medicine Hospital, Department of Otorhinolaryngology Head and Neck Surgery, Yan’an, China

**Keywords:** Allergic rhinitis, Posterior nasal neurectomy, Minimally invasive surgical procedures, Measurement

## Abstract

**Objective:**

This study aims to analyze how to perform a more minimally invasive and precise excision of posterior nasal neurectomy to reduce surgical time, minimize surgical trauma, and evaluate the clinical efficacy of this procedure in the treatment of moderate to severe persistent allergic rhinitis.

**Methods:**

This study included a total of 110 patients, randomly divided into an experimental group and a control group based on whether preoperative CT three-dimensional positioning was used and whether middle turbinate mucosal graftcoverage was applied during surgery. In the experimental group, preoperative CT measurements were used to locate the sphenopalatine foramen, and intraoperatively, the accuracy of the positioning method was verified. Middle turbinate mucosal graftcoverage was applied to shorten the postoperative recovery time. Both groups were evaluated using Visual Analog Scales (VAS) and Rhinoconjunctivitis Quality of Life Questionnaire (RQLQ) to compare the differences in symptoms between baseline and six months after operation. Regular endoscopic examinations were conducted to observe the status of mucosal epithelialization at the surgical site.

**Results:**

Preoperative CT measurements provided accurate intraoperative localization of the sphenopalatine foramen, allowing for a smaller surgical wound size. The average distance from the sphenopalatine foramen to the posteroinferior end of the uncinate process was 20.25 ± 2.69 mm, and from the sphenopalatine foramen to the upper edge of the inferior turbinate, it was 11.05 ± 1.71 mm. There was no statistically significant difference in preoperative symptom scores between the two groups (*p* > 0.05). At six months after operation, there were significant differences in VAS scores compared to baseline in both the experimental and control groups (*p* < 0.05). The average time for mucosal graftsurvival in the experimental group was 3.5 ± 1.0 weeks, while in the control group, the average time for exposed bone surface epithelialization was 9.0 ± 1.5 weeks.

**Conclusion:**

Preoperative CT measurements for locating the sphenopalatine foramen and the use of middle turbinate mucosal grafts in the posterior nasal neurectomy are safe and effective. This new procedure reduces surgical wound size, shorten surgical time, accelerate postoperative wound recovery, and could possibly reduce complications of bleeding after surgery.

**Level of evidence:**

Level 3.

## Highlights

• Minimally invasive procedure of posterior nasal neurectomy.
• Accurate and cost-effective way to locate the sphenopalatine foramen.
• Quicker postoperative wound recovery and fewer surgical complications.

## Introduction

The surgical treatment is a second-line approach for allergic rhinitis.^1^ With the rising incidence of allergic rhinitis, more patients with moderate to severe persistent cases are seeking treatments beyond medication.^2^ Previous research has confirmed that posterior nasal neurectomy has good postoperative efficacy, especially within 3–5 years.^3^, ^4^ For eligible patients, the main concerns when choosing posterior nasal neurectomy include surgical trauma, postoperative recovery time, and potential postoperative complications such as nasal bleeding. This issue is equally challenging for surgeons. This study aims to introduce a new surgical method that utilizes precise preoperative imaging planning, reduces mucosal loss during surgery, and employs autologous mucosal grafts to repair the surgical cavity.

## Methods

### Sample size calculation

Based on a preliminary study^6^ (using the nasal/eye symptoms in the RQLQ as the main indicator, with scores of 9.64 ± 1.98 in the experimental group and 8.93 ± 2.87 in the control group), we calculated that a sample of 75 patients (50 in the experimental group and 25 in the control group) would provide the study with 80% power to detect non-inferiority at a one-sided alpha of 0.05. The margin of non-inferiority was 0.8. Considering an anticipated dropout rate of 5%, the total sample size required is 81 (54 in the experimental group and 27 in the control group).

### Patients

All participants signed an Informed Consent document prior to participation, which received ethical approval from The Committee on Medical Ethics of our hospital (Ethical approval number: S2023249). The study inclusion criteria were: (1) All patients met the diagnostic criteria for perennial allergic rhinitis as outlined in the Chinese Guidelines for the Diagnosis and Treatment of Allergic Rhinitis (2022, revised edition), and serum IgE testing indicated sensitization to perennial allergens such as dust mites; (2) All patients had received standard pharmacological treatment but did not achieve satisfactory therapeutic outcomes or were unable to tolerate drug therapy or specific immunotherapy.

Patients were excluded from the study if they met any of the following criteria: (1) Patients who had not previously received standard drug therapy or specific immunotherapy; (2) Patients unable to tolerate surgical treatment including uncontrolled asthma, coagulation disorders and acute infections; (3) Patients with documented mental disorders or poor compliance; (4) Patients not able to accept the potential complications and risks associated with surgical treatment; (5) Patients who had undergone previous nasal nerve surgery; (6) Patients with evidence of sinusitis, nasal cysts, or any cancers of the nasal cavity or sinuses upon sinonasal Computed Tomography (CT) assessment.

### Randomization

The total of 110 patients enrolled in this study who had been diagnosed with AR for over 1 year at the time of diagnosis and were randomly assigned in a ratio of 2 to 1 to either the experimental group or the control group through simple randomization. In the control group, 30 patients underwent conventional surgery for posterior nasal neurectomy. In the experimental group, 80 patients underwent preoperative measurement and localization of the sphenopalatine foramen using three-dimensional CT reconstruction, and middle turbinate mucosal grafts were used to cover the exposed bone around the sphenopalatine foramen. All patients underwent endoscopic examination and sinus CT scans before surgery and completed both VAS and RQLQ assessments for nasal symptoms to establish baseline measurement.

### Localization of the sphenopalatine foramen

**Fig. 1:**
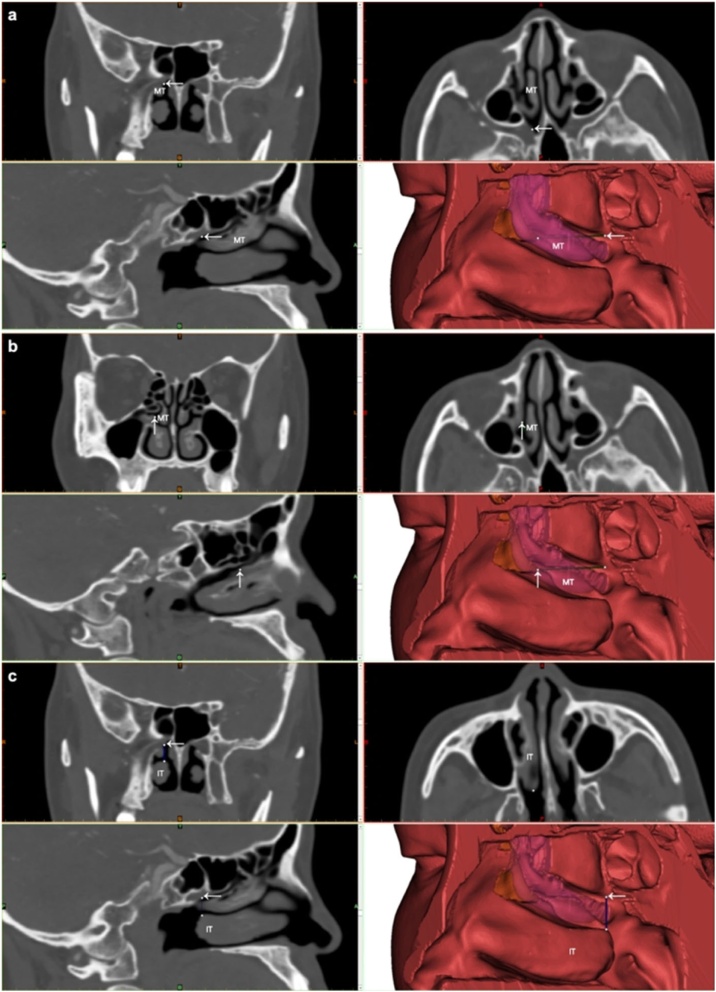
Three-dimensional reconstruction and measurement of the sphenopalatine foramen in the experimental group using mimics software on sinus CT. (a) CT scan and three-dimensional reconstruction illustrating the location of the sphenopalatine foramen. (b) CT and three-dimensional reconstruction showing the position of the posteroinferior end of the uncinate process and the spatial distance to the sphenopalatine foramen. (c) Measurement of the vertical distance from the sphenopalatine foramen to the upper edge of the inferior turbinate. MT, Middle Turbinate; IT, Inferior Turbinate; White ←, Sphenopalatine foramen; White ↑, Posteroinferior end of the uncinate process. In the 3D reconstruction, the pink structure represents the semi-transparent middle turbinate, the orange structure represents the uncinate process, the green line indicates the spatial distance from the sphenopalatine foramen to the posteroinferior end of the uncinate process, and the blue line represents the vertical distance from the sphenopalatine foramen to the upper edge of the inferior turbinate.

In previous literature, methods for localizing the sphenopalatine foramen have been relatively crude. Due to the considerable variability in the location of the sphenopalatine foramen, it cannot be precisely located using a single constant anatomical landmark. In the experimental group, we primarily used the posteroinferior end of uncinate process and the upper edge of the inferior turbinate as markers. Prior to surgery, the location of the sphenopalatine foramen was identified and marked by reviewing axial, coronal, and sagittal sinus CT images, as shown in Fig. 1a. The posteroinferior end of the uncinate process was also located and marked, as shown in Fig. 1b. Using Mimics (version 22.0, Materialise, Leuven, Belgium) software, a three-dimensional reconstruction of the lateral wall structures of the nasal cavity was created, and measurements were taken using the measuring tools to determine the distance between the sphenopalatine foramen and the posteroinferior end of the uncinate process, as seen in Fig. 1b. Additionally, the vertical distance between the sphenopalatine foramen and the upper edge of the inferior turbinate was measured, as shown in Fig. 1c. This enabled us to locate the sphenopalatine foramen more precisely, allowing the incision to be made as close as possible to the foramen while maintaining safety during the procedure. This approach helps reduce tissue loss and minimize surgical trauma.

### Surgery

All surgeries in this study were performed by the same surgeon, using a rigid endoscope (0 °, 4 mm; Karl Storz, Tuttlingen, Germany) under general anesthesia. In the control group, the surgery involved vertically excising the lower one-third of the middle turbinate and made a vertical incision to the bone surface in the middle-lower part of the middle meatus. We then separated the mucosal graft, exposing the perpendicular plate of the palatine bone, the ethmoidal crest, and the orbital process. We located the sphenopalatine foramen and the vascular nerve bundle passing through it. Using a coblation device, we completely ablated the mucosa and submucosal vascular nerve bundle within 1 cm around the sphenopalatine foramen down to the bone surface. The wound was covered with a gelatin sponge, and the procedure was completed after nasal cavity packing with Merocel nasal sponges.

**Fig. 2:**
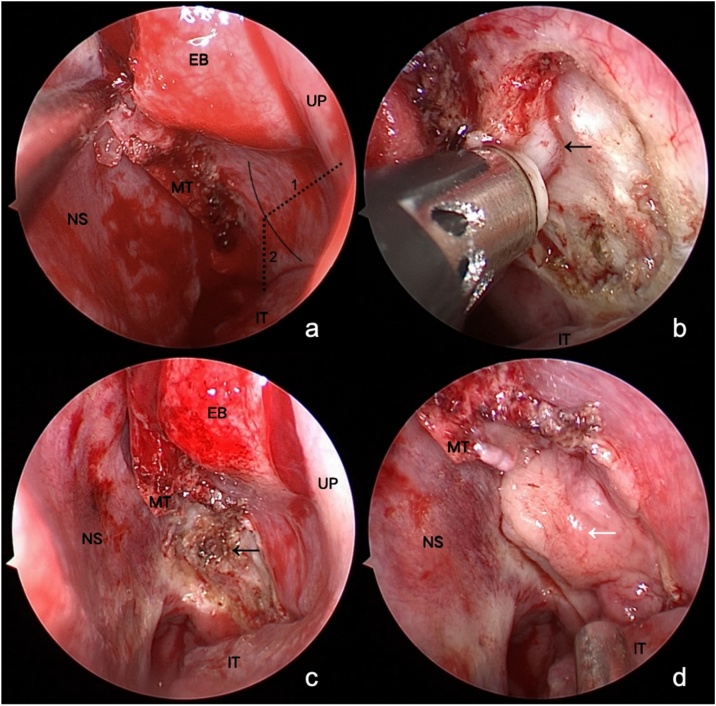
Surgical steps in the experimental group. (a) Removal of the lower one-third of the vertical portion of the middle turbinate and precise localization of the sphenopalatine foramen. Dotted line 1 indicates the distance from the posterior-inferior end of the uncinate process to the sphenopalatine foramen, and dotted line 2 shows the distance from the sphenopalatine foramen to the upper edge of the inferior turbinate. (b) Ablation of the mucosa and soft tissues using a coblation device, approximately 5 mm anterior and inferior to the localized point, exposing the sphenopalatine foramen. (c) Complete ablation of the soft tissues within a 5 mm radius around the sphenopalatine foramen, down to the bone surface, creating an “island-like” appearance of the vascular nerve bundle. The soft tissues emerging from the sphenopalatine foramen are also ablated. (d) Coverage of the sphenopalatine foramen and exposed bone surface with a free mucosal graft from the middle turbinate. Biological glue is used for fixation. UP, Uncinate Process; EB, Ethmoid Bulla; MT, Middle Turbinate; IT, Inferior Turbinate; NS, Nasal Septum; ←, Vascular nerve bundle around the sphenopalatine foramen; White ←, Mucosal graft of the middle turbinate.

In the experimental group surgery, we improved the conventional surgical approach. After vertically excising the lower one-third of the middle turbinate, we preserved the mucosal graft. Using numerical values obtained through preoperative CT localization measurements, we located the sphenopalatine foramen based on the posteroinferior end of the uncinate process and the upper edge of the inferior turbinate. Using a coblation device, we ablated the soft tissues 5 mm anterior and inferior to the localization point. We pushed and separated the mucosa backward and upward to further confirm the position of the sphenopalatine foramen. With the sphenopalatine foramen as the center, we thoroughly ablated the soft tissues within a 5 mm radius around it down to the bone surface, making the vascular nerve bundle around the sphenopalatine foramen “island-like”. We further used the coblation device to treat the soft tissues emerging from the sphenopalatine foramen. We trimmed the free mucosal graft of the middle turbinate and used it to cover the sphenopalatine foramen and the exposed bone surface around it. Similar to skin grafting, it was essential to create 2–3 perforations on the mucosal graft's surface to prevent submucosal hematoma and graft detachment. We fixed the mucosal graft with biological glue and completed the whole procedure after nasal packing (see Fig. 2).

Depending on the patient’s postoperative recovery, saline nasal irrigation and intranasal corticosteroids (budesonide nasal spray) were used as postoperative treatment during the first 1–3 months after surgery. The duration of postoperative treatment was determined by the severity of symptoms and the condition of the mucosa. Regular nasal endoscopic examinations and nasal cavity cleaning were performed.

### Assessment of treatment efficacy

Patient self-assessment scores were used to evaluate the severity of symptoms using both the VAS and RQLQ. Symptom scores ranged from 0 to 10 (0 = No symptoms; 10 = Most severe discomfort). Additionally, patients underwent regular nasal endoscopic examinations to observe postoperative recovery and to record any complications that occurred during the postoperative period.

### Data analysis

All statistical analyses in this study were conducted using SPSS 27.0 (IBM Corporation, NY, USA). The normality of the data was assessed using the Shapiro-Wilk test. Continuous data were presented as mean ± Standard Deviation (SD). For normally distributed data, values between the experimental group and the control group were compared using independent samples *t*-tests, while values within each group over time were compared using paired samples *t*-tests. For non-normally distributed data, the Wilcoxon signed-rank test was used for comparisons. Comparisons of categorical variables were performed using the Chi-Square test, with *p* < 0.05 indicating statistical significance.

## Results

### Baseline patient characteristics

A total of 110 patients were included in the study, with 80 patients in the experimental group and 30 patients in the control group. All patients completed VAS questionnaires at baseline and 6 months postoperatively. The baseline demographic characteristics and clinical outcomes of the study patients are presented in Table 1. There were no statistically significant differences between the experimental and control groups in terms of patient age, gender, duration of Allergic Rhinitis (AR), and preoperative VAS scores (Table 1) (*p* > 0.05).

**Table 1:** Demographic and clinical information and nasal symptom VAS scores in the experimental and control groups.

Variable	Experimental group	Control group
Number of patients	80	30
Gender (male / female)	47 / 33	6 / 24
Age (years, mean ± SD)	34.11 ± 9.31	35.34 ± 8.22
Duration of AR (years, mean ± SD)	3.75 ± 1.47	3.56 ± 1.89
Total VAS (mean ± SD)	14.31 ± 0.84	14.00 ± 1.47
Nasal Oobstruction VAS	5.31 ± 0.32	5.13 ± 0.42
Rhinorrhea VAS	3.74 ± 0.29	3.57 ± 0.49
Sneezing VAS	3.00 ± 0.34	2.90 ± 0.51
Nasal itching VAS	2.26 ± 0.27	2.40 ± 0.55

### CT measurement results of the sphenopalatine foramen

The measurements showed an average distance of 20.25 ± 2.69 mm from the sphenopalatine foramen to the posteroinferior end of the uncinate process, and an average distance of 11.05 ± 1.71 mm from the sphenopalatine foramen to the upper edge of the inferior turbinate. No significant differences were found between the two groups in either the distance from the sphenopalatine foramen to the posteroinferior end of the uncinate process or the distance from the sphenopalatine foramen to the upper edge of the inferior turbinate (*p* > 0.05).

### Changes in the main postoperative symptoms

**Fig. 3:**
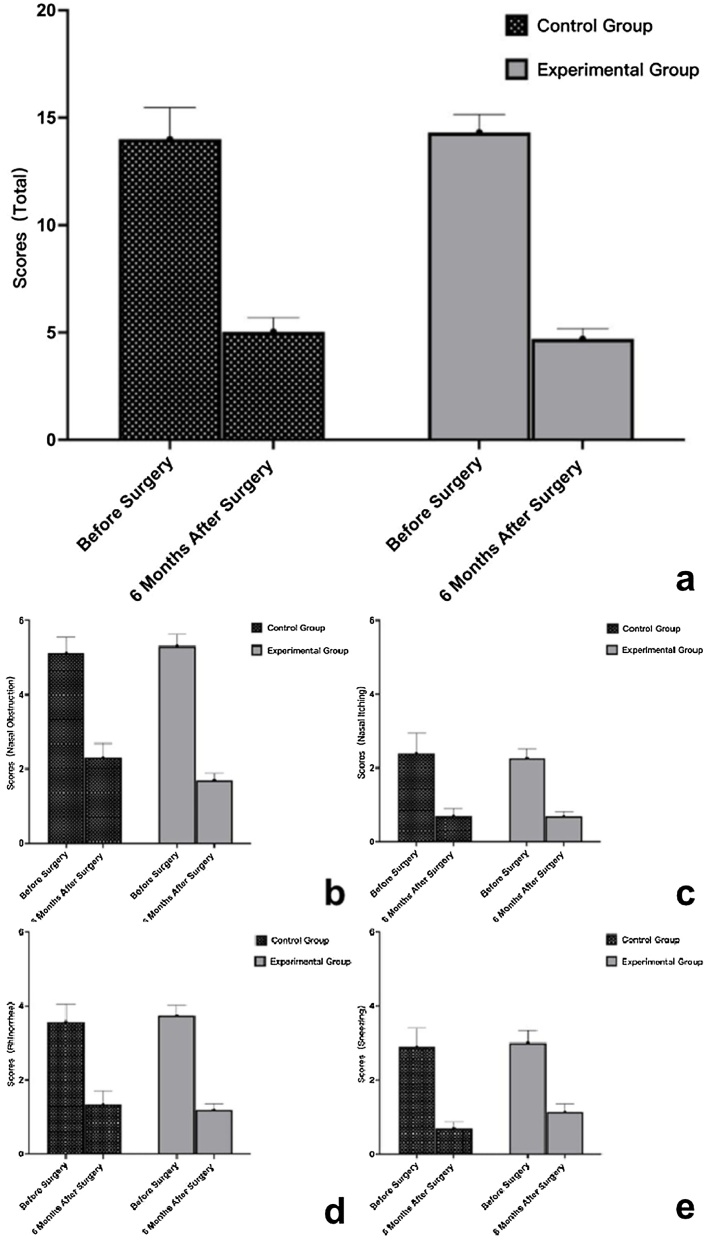
Comparison of nasal symptom VAS scores before and 6-months after surgery in the experimental and control groups (a) Total scores; (b) Nasal obstruction; (c) Nasal Iitching; (d) Rhinorrhea; (e) Sneezing.

The VAS and RQLQ scores for nasal symptoms during the follow-up period for both groups are shown in Table 2, Table 3 and Fig. 3. We observed that there were significant differences (*p* < 0.05) in the VAS and RQLQ scores for nasal symptoms relative to baseline in both the experimental group and the control group at 6 months after surgery. There were significant differences (*p* < 0.05) in the RQLQ scores in postoperative symptoms and quality of life between the two groups, and the experimental group had relatively lower scores of RQLQ compared with the control group.

**Table 2:** Comparison of nasal symptom VAS in the experimental and control groups before and 6 months after surgery.

Variable	Experimental group	Control group	*p-*value
Nasal obstruction (before surgery)	5.31 ± 0.32	5.13 ± 0.42	0.761
Nasal obstruction (6-months after surgery)	1.69 ± 0.20	2.30 ± 0.39	0.134
Rhinorrhea (before surgery)	3.74 ± 0.29	3.57 ± 0.49	0.763
Rhinorrhea (6-months after surgery)	1.19 ± 0.16	1.33 ± 0.38	0.682
Nasal itching (before surgery)	2.26 ± 0.27	2.40 ± 0.55	0.805
Nasal itching (6-months after surgery)	0.69 ± 0.14	0.70 ± 0.19	0.961
Sneezing (before surgery)	3.00 ± 0.34	2.90 ± 0.51	0.876
Sneezing (6-months after surgery)	1.14 ± 0.22	0.70 ± 0.17	0.248
Total score (before surgery)	14.31 ± 0.84	14.00 ± 1.47	0.849
Total score (6-months after surgery)	4.70 ± 0.49	5.03 ± 0.67	0.711

**Table 3:** Comparison of RQLQ in the experimental and control groups before and 6 months after surgery.

Variable	Experimental group	Control group	*p-*value
Nasal symptoms (before surgery)	15.98 ± 5.05	15.11 ± 5.29	0.203
Nasal symptoms (6-months after surgery)	5.05 ± 3.51	8.73 ± 5.13	<0.001
Eye symptoms (before surgery)	13.30 ± 6.49	12.54 ± 6.36	0.276
Eye symptoms (6-months after surgery)	3.63 ± 2.80	7.13 ± 5.56	<0.001
NNES (before surgery)	21.20 ± 10.16	21.17 ± 9.98	0.494
NNES (6-months after surgery)	7.00 ± 5.91	12.46 ± 9.13	<0.001
Sleeping (before surgery)	11.68 ± 4.60	10.57 ± 4.86	0.123
Sleeping (6-months after surgery)	4.00 ± 3.31	6.23 ± 4.31	0.003
Others (before surgery)	37.68 ± 10.13	37.21 ± 12.08	0.420
Others (6-months after surgery)	12.53 ± 7.73	22.83 ± 13.76	<0.001
Total score (before surgery)	87.03 ± 23.55	85.00 ± 27.93	0.350
Total score (6-months after surgery)	29.05 ± 17.96	51.89 ± 30.77	<0.001

### Postoperative surgical cavity recovery

Regular nasal endoscopy examinations and nasal cavity cleaning were performed on both groups of patients postoperatively to observe the recovery of the surgical areas. It was found that there was crusting covering the surgical area in both groups one week after surgery. The average time for the epithelialization of the exposed bone surface in the control group was (9.0 ± 1.5) weeks, while in the experimental group, the average time for mucosal graft survival was (3.5 ± 1.0) weeks.

### Complications and mortality rate

All patients were discharged approximately 3 days after surgery, with no perioperative mortality. There were no major postoperative complications, such as orbital nerve or cranial nerve injury, significant nasal bleeding, palatal numbness, or persistent dry eye syndrome. In the control group, two cases experienced unilateral nasal bleeding around 1 month postoperatively, and nasal endoscopic examination revealed bleeding in the sphenopalatine foramen area, suspected to be from the transected end of the posterior lateral nasal artery. After hemostasis was achieved using electrocautery under nasal endoscope, there was no further bleeding.

## Discussion

Throughout, there has been significant controversy regarding the surgical treatment of allergic rhinitis. Overall, for patients with moderate to severe persistent allergic rhinitis, surgical intervention primarily serves as an alternative when pharmacological treatments yield unsatisfactory results. Current literature generally acknowledges the definitive relief of allergic rhinitis symptoms through high-selective resection surgery of the vidian nerve trunk or its branches,^5^ although there may be a risk of reduced efficacy in the medium to long term. Some studies have also found long-term effectiveness and safety of the surgery.^6^ Japanese scholars have conducted an 8-year follow-up study, revealing mild adverse events associated with symptom improvement 8 years after surgery.^7^ Some experts who previously did not endorse this surgery believe that the invasiveness of surgical procedures, uncertainty regarding long-term efficacy, and potential postoperative complications may not justify the associated risks. They may refrain from performing this surgery themselves or assert that the current evidence in the medical literature is of low quality. As a center that performs this surgery, we believe that with the continuous advancement of high-definition endoscopic technology, a deeper understanding of neuroanatomy, and proactive prevention of postoperative complications, surgical trauma has been greatly reduced, and the postoperative recovery time has significantly shortened, leading to increased patient satisfaction.

This study included 110 patients who underwent posterior nasal neurectomy. Short-term efficacy showed significant improvements in nasal obstruction, rhinorrhea, nasal itching, and sneezing. Nasal obstruction improved the most, followed by rhinorrhea, demonstrating the procedure’s efficacy. Quality of life also improved. In the experimental group, a mucosal graft was used to repair the vascular nerve plexus incision site. It was found that the mucosal graft revascularized within 2–3 weeks after surgery, significantly reducing the occurrence of local bleeding and scab formation. The production, transplantation, and fixation methods of the mucosal graft have a significant impact on postoperative survival. During the application of mucosal graft technique, we found that partial submucosal tissue reduction of the middle turbinate was necessary. The size of the exposed bone surface in the nasopharyngeal nerve ablation area was measured, and the mucosal graft was cut to an appropriate size. After thoroughly clearing nasal cavity blood accumulation, the mucosal graft was applied, and a small amount of biological glue was used for fixation. This surgical method was employed in all cases in the experimental group, and both mucosal grafts were well maintained postoperatively.

Precise localization of the sphenopalatine foramen during surgery helps minimize surgical trauma. Specifically, a careful review of preoperative imaging to determine the relative relationship between the sphenopalatine foramen's location and the root of the middle turbinate is helpful for targeting the ablation as close as possible to the posterior nasal nerve. Based on previous literature, most methods for locating the sphenopalatine foramen rely on making a vertical incision at the posterior fontanel, peeling the mucosa backward to expose the ethmoidal crest and using it as an anatomical landmark to locate the sphenopalatine foramen.^6^, ^8^ This method has some blind spots and relatively greater trauma. In this study, three-dimensional reconstruction of the patient’s CT scan was performed preoperatively to locate the posteroinferior end of the uncinate process, the sphenopalatine foramen, and the upper edge of the inferior turbinate. During surgery, based on the preoperatively measured data, the sphenopalatine foramen position was precisely located after excision of the vertical part of the middle turbinate, using the coblation device tip with a diameter of 3 mm. This not only reduces unnecessary mucosal loss but also avoids damage to the main branch of the sphenopalatine artery. A 360-degree circumferential ablation of the tissues surrounding the sphenopalatine foramen ensured thorough ablation and cutting of the small vascular nerve bundle passing through the accessory foramen, thereby maximizing the surgical effect and preventing peripheral nerve regeneration.^9^, ^10^

This study found no significant difference in postoperative efficacy between the experimental and control groups, with both showing significant improvements compared to preoperative symptoms, consistent with previous literature.^11^, ^12^ In the group without mucosal grafts, epithelialization took nearly 3 months, and regular nasal endoscopy was required for cleaning the nasal cavity. Exposed bone surfaces were prone to scabbing, and nasal secretions could obstruct this area, causing discomfort that was difficult to alleviate even with nasal irrigation. In contrast, the experimental group, which used mucosal grafts, achieved revascularization within 3–4 weeks after surgery and maintained a relatively clean nasal cavity through daily irrigation and removal of scabs under endoscopy.

Although, in general, the incidence rate of complications associated with this surgery is relatively low, surgeons who intend to perform this procedure should nonetheless emphasize the meticulous manipulation of each individual case and strive to minimize the occurrence of complications, such as orbital or cranial nerve injuries, massive nasal hemorrhage, palatal numbness, or persistent dry eyes. In this study, two patients in the control group experienced postoperative bleeding, which required endoscopic examination and electrocautery treatment. The bleeding originated from small arterial branches at the end of the posterior nasal artery and was successfully controlled. No bleeding occurred in the experimental group. Nasal bleeding is a common complication of posterior nasal neurectomy, often involving small arteries that pose a risk of rapid blood loss and aspiration. Effective hemostasis requires expertise and appropriate equipment, such as posterior nasal packing or electrocautery. Reducing bleeding complications is important for ensuring patient safety.

In summary, the use of precise preoperative measurement and the mucosal graft technique can make posterior nasal neurectomy more precise and minimally invasive. This approach minimizes mucosal loss, accelerates epithelialization, and improves recovery quality. However, the technique does require careful surgical planning, may increase operative time, and could incur higher costs due to the use of specialized instruments and procedures. While this minimally invasive approach may offer benefits for patients with moderate to severe allergic rhinitis unresponsive to pharmacological treatment, these factors should be weighed against other available treatment options.

## Conclusion

Posterior nasal neurectomy effectively alleviates rhinorrhea, nasal obstruction, nasal itching and sneezing in patients with allergic rhinitis, and improves quality of life, demonstrating favorable short-term postoperative efficacy. Thorough preoperative radiological examination aids in reducing intraoperative incisions, while the application of mucosal graft technique during surgery accelerates postoperative epithelialization, thereby contributing to a lower incidence of bleeding complications and an improved quality of life during the postoperative recovery period.

## Ethics committee approval

This research obtained ethical approval from Peking University third hospital’s ethics committee (Approval nº S2023251).

## Funding

Shan xi Natural Science Foundation (2021JQ-942).

Research Project of Beijing Chronic Disease Prevention and Health Education Research Association (BJMB0012021025021).

## Declaration of competing interest

The authors declare no conflicts of interest.
